# Quality of Life Improvement Through Endoscopic Stent Placement for Cancer-Related Intestinal Obstruction: A Report of Two Cases

**DOI:** 10.7759/cureus.92033

**Published:** 2025-09-11

**Authors:** Hironobu Yanagie, Yasumasa Nonaka, Shinichiro Goto, Naoto Nishinari, Yoshitaka Furuya

**Affiliations:** 1 Department of Surgery, Keiaikai Kojin Hospital, Morioka, JPN; 2 Department of Surgery, Keiaikai Houyou Hospital, Hanamaki, JPN; 3 Institute of Engineering Innovation, School of Engineering, The University of Tokyo, Tokyo, JPN; 4 Department of Medical Technology, Niigata University of Pharmacy and Medical and Life Sciences, Niigata, JPN; 5 Department of Surgery, Sodegaura Satsukidai Hospital, Sodegaura, JPN

**Keywords:** colon cancer, endoscopic procedure, intestinal obstruction by cancer, pancreas cancer, qol improvement, self-expandable metal stent

## Abstract

Quality of life (QOL) is often severely reduced in cancer patients due to a decline in overall health and the occurrence of ileus caused by cancer-related intestinal obstruction. We report two cases in which QOL was improved through endoscopic stent placement (ESP). A 94-year-old woman with an 8-cm circumferential tumor in the ascending colon complicated by ileus underwent ESP, during which the intestinal lumen was secured with a 12-cm stent using the double-stenting technique. Oral intake resumed after the procedure, and she was discharged home, both of which contributed to improved QOL. Similarly, an 83-year-old woman with pancreatic head cancer began vomiting three months after choledochojejunostomy and underwent ESP. The pancreatic tumor had invaded from the duodenal bulb to the descending part of the duodenum, and the stent was positioned with the distal end in the lower descending duodenum and the proximal end in the prepyloric region of the stomach. Her QOL improved, and she was discharged home. ESP may effectively relieve ileus and facilitate the resumption of oral intake. Therefore, we actively consider this technique for managing cancer-related intestinal obstruction in advanced cases to improve QOL.

## Introduction

In advanced cancer cases, patients often experience a marked decline in quality of life (QOL) due to the progressive deterioration of overall health, compounded by ileus resulting from cancer-related intestinal obstruction.

Endoscopic stent placement (ESP) has become a well-established approach for the management of malignant colorectal obstruction [1-7]. Dohmoto was the first to report the use of a self-expandable metal stent (SEMS) as a palliative intervention for stenosis caused by rectal cancer invasion [8]. Previous studies have demonstrated the utility of ESP in obstructive colorectal cancer, particularly for facilitating one-stage resection or laparoscopic surgery. By providing preoperative decompression in cases of acute colorectal obstruction, ESP enables the resumption of oral intake before surgery, temporary hospital discharge, and the possibility of an elective surgical intervention without the need for stoma creation [4,5].

The European Society of Gastrointestinal Endoscopy has raised some concerns regarding the efficacy of ESP in patients with colorectal cancer-related ileus [9]. Although colostomy remains a commonly employed definitive intervention, ESP is now widely recommended as a palliative alternative. Consequently, ESP is increasingly being adopted in place of colonic stomas in palliative care, as it offers lower complication rates than colostomy [4].

Pancreatic cancer carries a high mortality rate, with approximately 85% of patients presenting with unresectable tumors due to extensive metastasis and invasion of adjacent structures [10,11]. Gastric outlet obstruction (GOO), often caused by duodenal invasion, is a late-stage complication of pancreatic cancer and occurs in up to 20% of cases. Traditionally, surgical gastroenterostomy has been the primary approach for managing GOO caused by pancreatic adenocarcinoma. However, growing evidence supports the use of ESP as an effective means of relieving obstruction and jaundice in patients with inoperable tumors [10-14].

ESP is now widely employed in the treatment of malignant gastrointestinal obstruction, either as a bridge to surgery or as a palliative intervention. We herein present two cases in which ESP, using a distal expansion method, improved QOL by relieving cancer-related intestinal obstruction.

This study was previously presented as a meeting abstract at the 2021 JDDW Annual Scientific Meeting on November 5, 2021.

## Case presentation

Case 1

**Figure 1:**
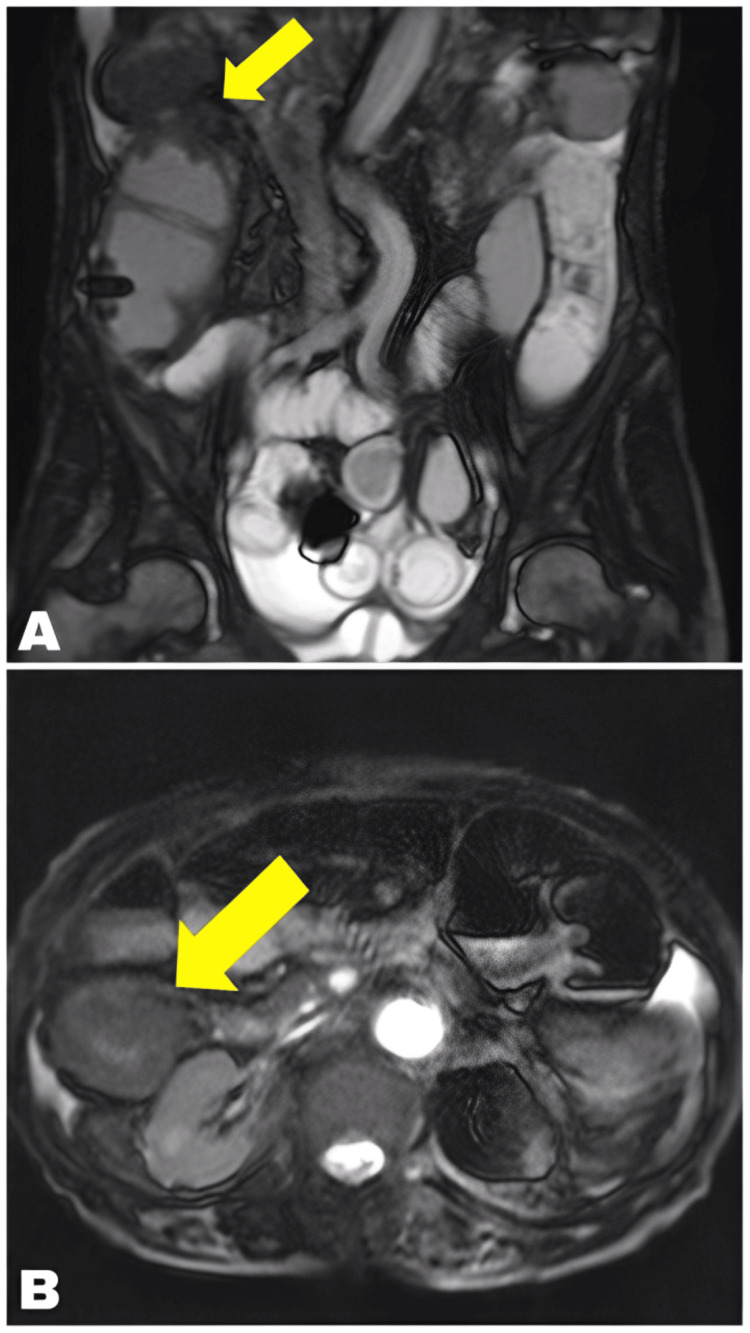
MRI of ascending colon cancer at admission MRI shows tumor occlusion and irregular wall thickening in the ascending colon near the hepatic flexure, along with proximal intestinal tract distension.

A 94-year-old woman was referred to our hospital in early April 2014 with abdominal pain and vomiting. MRI revealed proximal intestinal tract distension and irregular wall thickening in the ascending colon near the hepatic flexure (Figure 1). She was diagnosed with ileus and severe anemia, and ascending colon cancer was suspected. Continuous intravenous hyperalimentation and blood transfusion were initiated to stabilize her condition. As the patient declined surgical resection of the colon, ESP was performed in June 2014.

ESP

**Figure 2:**
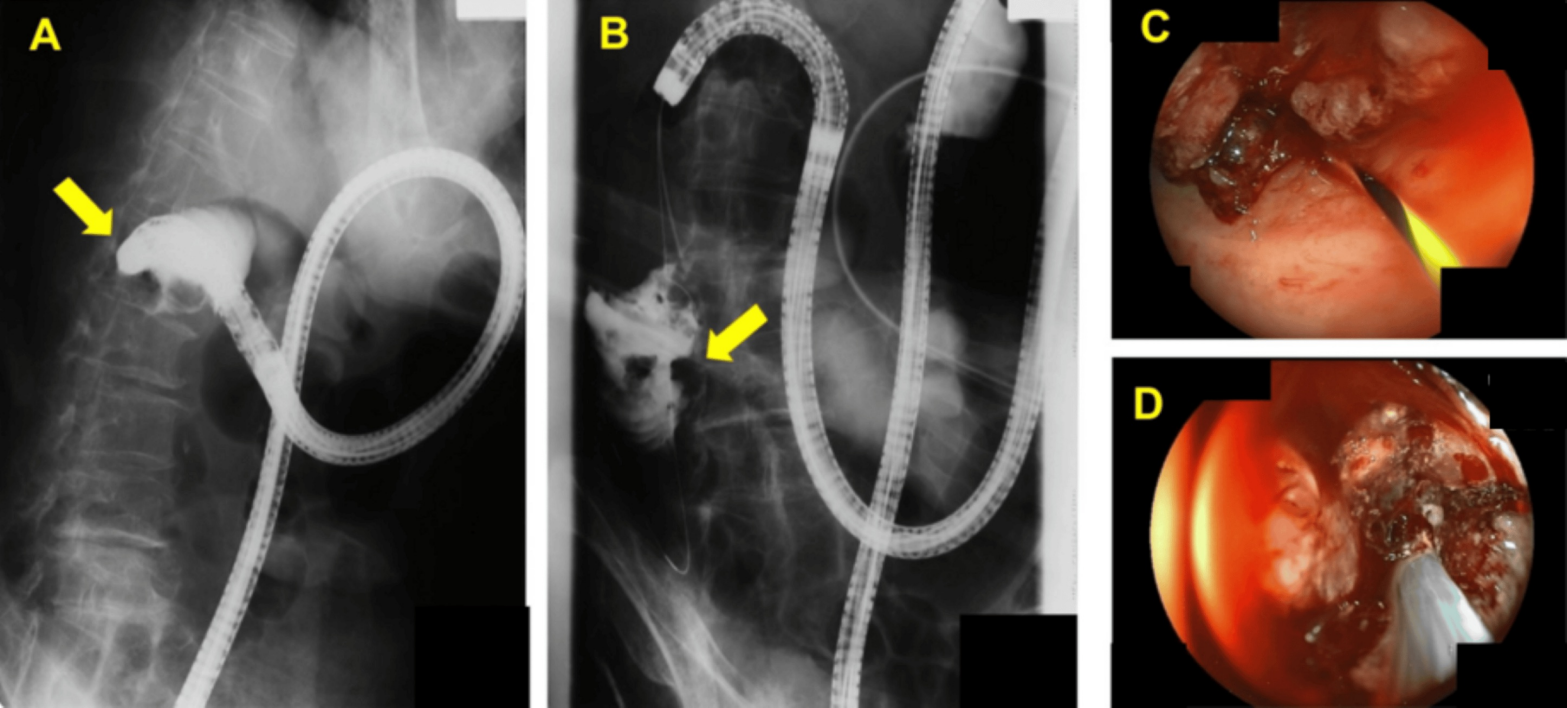
Tumor images obtained by colonoscopy and contrast imaging (A, B) A guidewire was inserted proximal to the tumor in the ascending colon using a colonoscope, followed by catheter insertion and contrast imaging. (C, D) Endoscopic findings showed a circumferential tumor.

**Figure 3:**
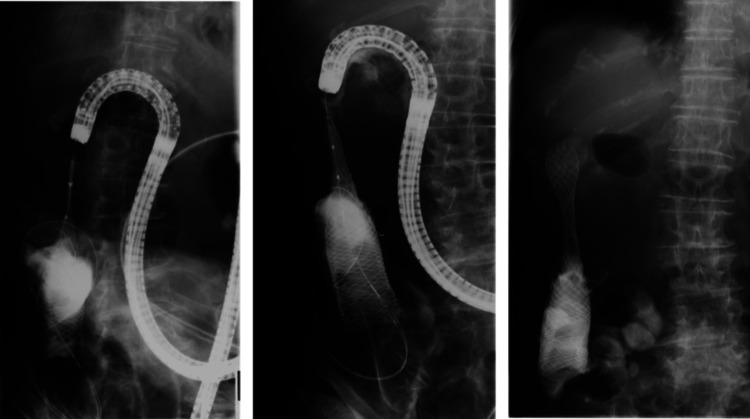
ESP in Case 1 A SEMS was placed. A WallFlex™ Colonic Stent (Boston Scientific Co., Ltd.) was inserted over a guidewire and positioned using the distal expansion method, with the safety margin estimated from the proximal end of the tumor. ESP, endoscopic stent placement; SEMS, self-expandable metal stent

An endoscopic examination revealed an approximately 8-cm circumferential tumor extending from the ascending colon to the hepatic flexure of the transverse colon (Figure 2). A colonoscope was advanced to the distal end of the tumor. A guidewire was then passed through the center of the tumor, followed by the insertion of a catheter, which enabled contrast imaging for improved visualization. Using the double-stenting technique, the lumen was secured with a 12-cm WallFlex™ Colonic Stent (Cat. No. 22300BZX0030800, Boston Scientific Co., Ltd., Marlborough, MA, USA) inserted over the guidewire (Figure 3). The distal portion of the stent was expanded with a safety margin from the proximal end of the tumor.

Post-procedure

**Figure 4:**
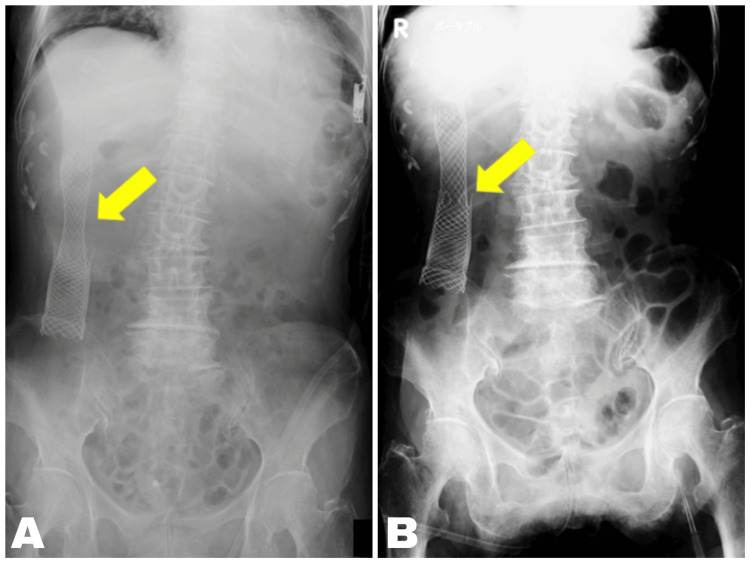
Post-procedure images of ESP in Case 1 Stent expansion was satisfactory, and oral intake subsequently resumed. (A) Day 10 after the procedure. (B) Day 47 after the procedure. ESP, endoscopic stent placement

Stent expansion was successful, and oral intake of a liquid diet resumed three days later (Figure 4). She was discharged two weeks after the procedure. Although the patient passed away from cancer three months later, she was able to eat a normal diet and spend her remaining time at home, engaging in daily activities according to her preferences. These factors contributed to an improved QOL.

Case 2

**Figure 5:**
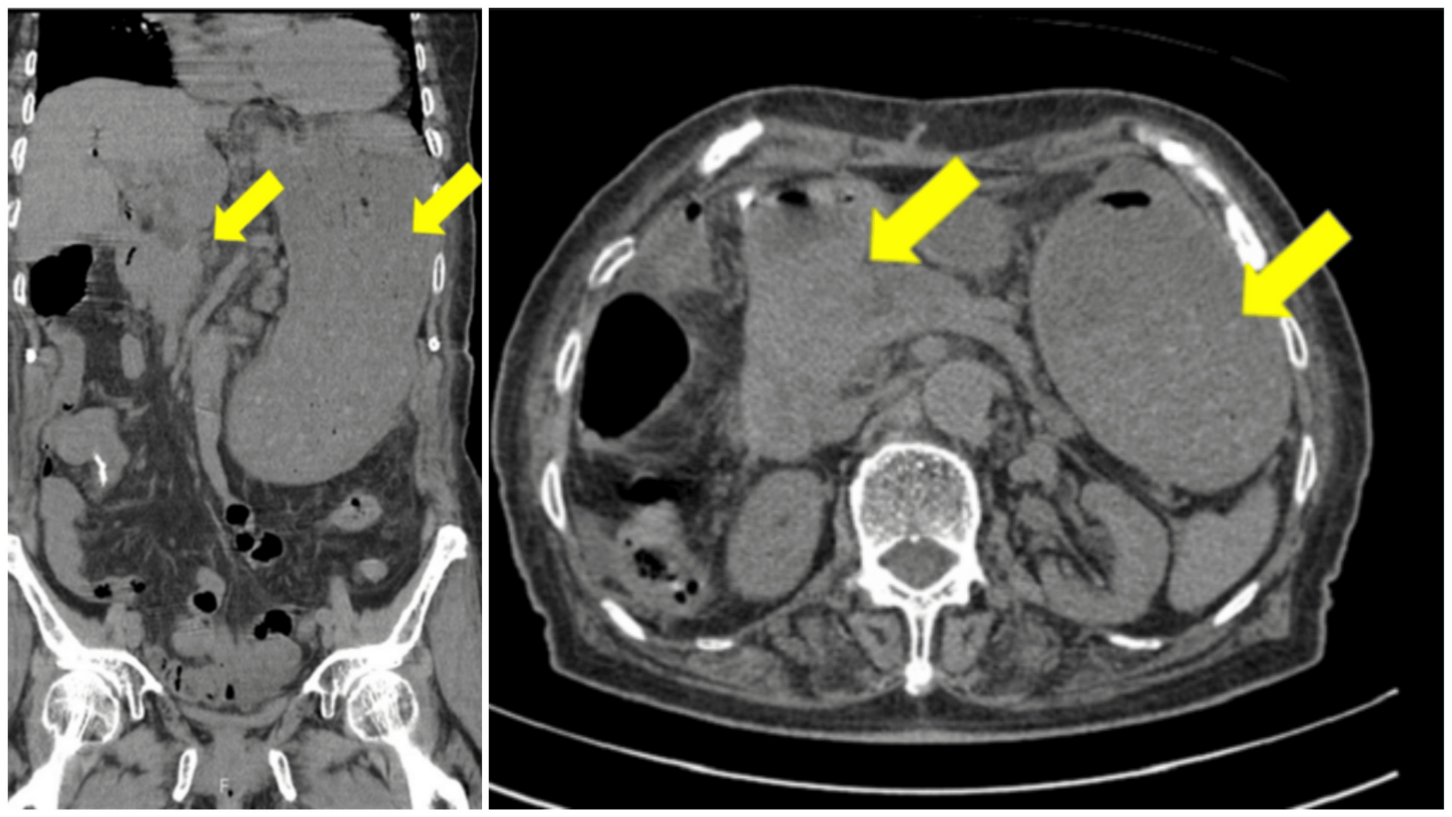
CT images of pancreatic head cancer at admission CT revealed tumor growth of the pancreatic head with duodenal invasion. Gastric distension due to duodenal obstruction was also observed.

An 83-year-old woman presented with obstructive jaundice and was diagnosed with pancreatic head cancer with portal vein invasion in February 2019. As the tumor was unresectable, bile drainage was performed via choledochojejunostomy in March 2019. However, she began vomiting in June 2019, and a CT revealed tumor growth with duodenal invasion (Figure 5).

ESP

**Figure 6:**
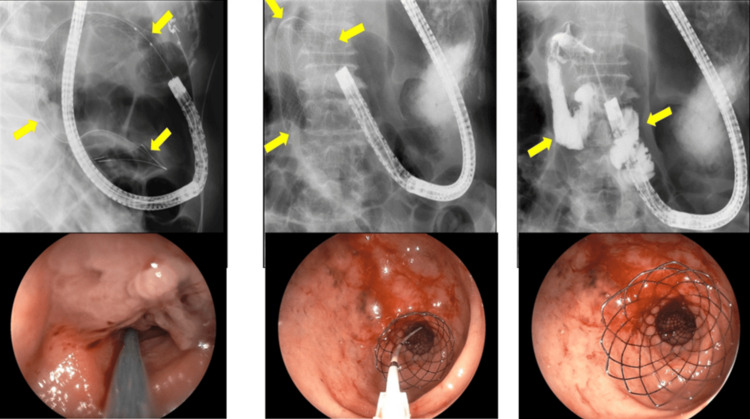
ESP in Case 2 A SEMS was placed. A WallFlex™ Duodenal Soft Stent (Boston Scientific Co., Ltd.) was inserted over a guidewire. The distal end of the stent was positioned in the lower descending duodenum, and the proximal end in the prepyloric region of the stomach, using the distal expansion method. ESP, endoscopic stent placement; SEMS, self-expandable metal stent

The patient underwent ESP in late June 2019. Endoscopic examination confirmed tumor invasion extending from the duodenal bulb to the upper portion of the descending duodenum. A WallFlex™ Duodenal Soft Stent (Cat. No: 22900BZX00029000, Boston Scientific Co., Ltd.) (Figure 6) was positioned with the distal end in the lower descending duodenum and the proximal end in the prepyloric region of the stomach. Stent expansion was satisfactory, and contrast imaging confirmed improved passage through the obstructed segment.

Post-procedure

**Figure 7:**
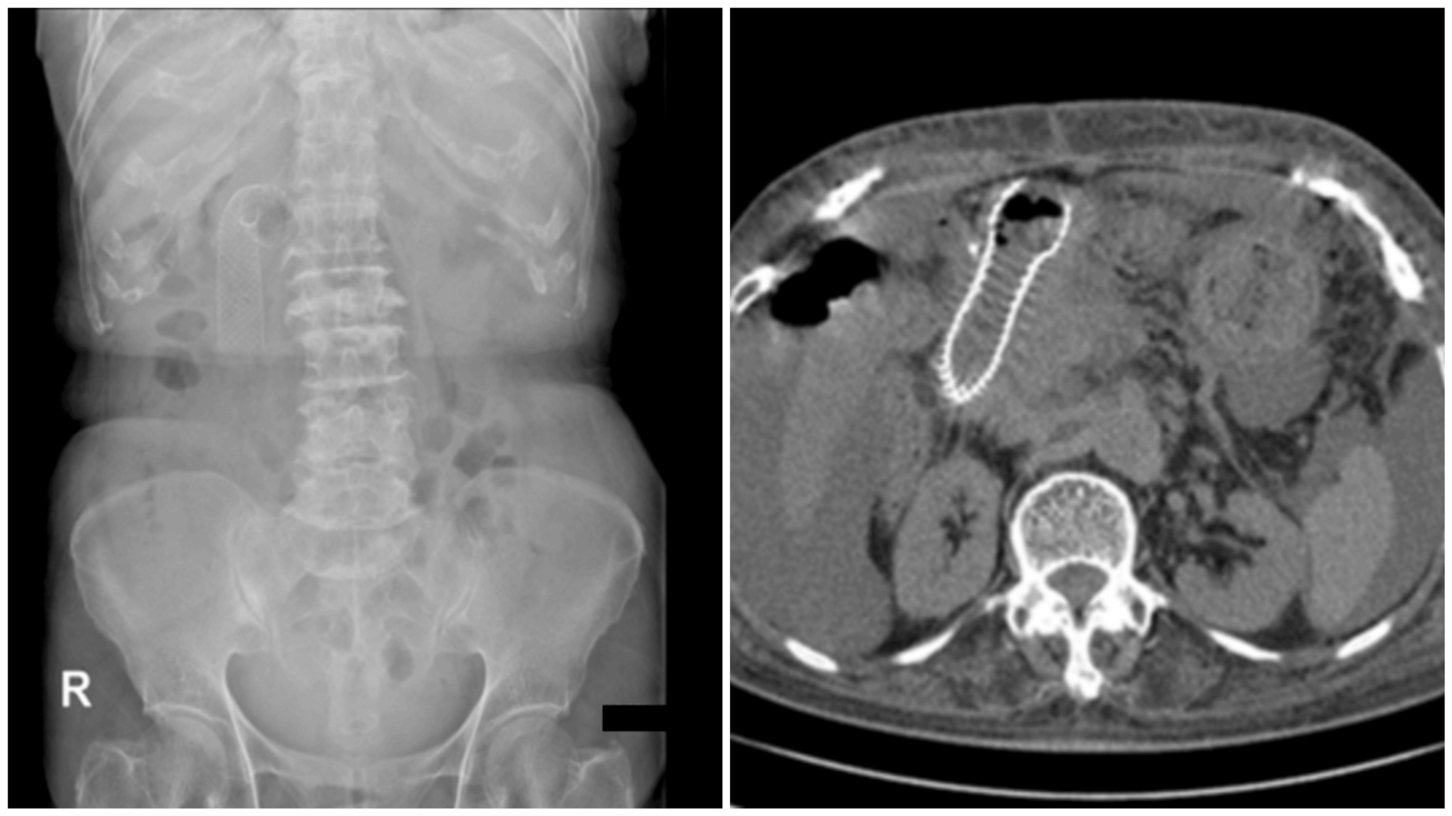
Post-procedure images of ESP in Case 2 Stent expansion was satisfactory, and oral intake resumed after the procedure. The patient was able to remain at home, which improved QOL. ESP, endoscopic stent placement; QOL, quality of life

The stent functioned effectively, and the patient resumed a liquid diet three days after the procedure (Figure 7). She was discharged six days later. Although she passed away from liver failure two months after the procedure, she was able to spend her remaining time at home, which contributed to an improved QOL.

## Discussion

In cases of colon cancer with intestinal obstruction, previous studies reported that patients who underwent ESP had shorter hospital stays and lower rates of intensive care unit admission compared with those who underwent surgery for cancer-related obstruction [4,6]. Common stent-related complications included colonic perforation, bleeding, stent migration, re-obstruction, and pain. Reocclusion was typically managed by stent replacement or the stent-in-stent technique. Regular follow-up every few months after ESP was strongly recommended to reduce the risk of reocclusion [4]. Multiple studies showed that the success rate of duodenal ESP exceeded 90% in selected patient populations, with severe complications such as stent migration and duodenal wall perforation being rare. Compared with gastrojejunostomy, ESP was associated with shorter hospital stays, faster resumption of oral intake, lower mortality rates, and reduced costs [1].

In pancreatic cancer, duodenal obstruction often occurs due to invasion by pancreatic head tumors or pancreatic body tumors involving the duodenojejunal junction. This obstruction caused symptoms such as nausea, vomiting, and poor oral intake, which significantly affected QOL and increased the risks of dehydration and malnutrition [10]. Sakai et al. reported a case in which ESP effectively relieved afferent loop obstruction caused by recurrent pancreatic cancer after pancreaticoduodenectomy [12].

The diagnosis of duodenal obstruction was typically confirmed by endoscopy, with contrast imaging performed to evaluate the length of the stricture and assess its suitability for stenting. Tumor staging was generally performed with CT. Reported complications included stent migration, duodenal perforation, and cholangitis, while reocclusion was commonly managed by the placement of additional stents [15].

Local photothermal heating under near-infrared laser irradiation was shown to suppress the formation of granulation tissue induced by ESP [16]. Furthermore, in an in vitro study, Delarue et al. demonstrated that compressive stress inhibited colon cancer cell proliferation by arresting the cell cycle in the G1 phase through overexpression of the cyclin-dependent kinase inhibitor p27kip1 [17,18].

ESP involved advancing a guidewire through the endoscope’s working channel and passing it beyond the obstruction. The selected stent needed to provide a 2-cm overlap on either side of the obstruction to ensure an adequate margin. Using the guidewire as a pathway, the SEMS system was positioned to proportionally cover the stenosis before deployment. Placement and luminal patency were confirmed both endoscopically and fluoroscopically [10].

In the two cases presented herein, tumors causing stenosis and obstruction were addressed by advancing a guidewire beyond the tumor to the opposite side. A catheter was then inserted via the guidewire, and contrast imaging was performed to delineate the tumor margins. To account for stent contraction and tumor growth, stent expansion was initiated from the distal end, ensuring that the stent edges extended more than 2 cm beyond the tumor margins. Saida emphasized the importance of considering the axial force of metallic stents, particularly when deploying enteral stents in curved sections of the gastrointestinal tract, as shortening rates vary among stents [19,20].

In these two cases, there was a significant improvement in the CROSS score, from 0 to 4, after stenting. The patients resumed drinking water on day 1 and oral intake on day 3 after ESP (Table 1) [3,6].

**Table 1:** Overview of our cases who underwent ESP as palliative treatment Score definitions: 0 = requiring continuous decompression; 1 = no oral intake; 2 = liquid or enteral nutrition; 3 = soft solid, low-residue, or full diet with symptoms of stricture; 4 = soft solid, low-residue, or full diet without symptoms of stricture [3,6]. CROSS, ColoRectal Obstruction Scoring System; ESP, endoscopic stent placement; PS, performance status

Age	Sex	Cancer	Stage	Stent	Complication	Stent patency (months)	PS (pre)	CROSS score (pre)	PS (post)	CROSS score (post)	Days to oral intake after ESP	Clinical course
94	F	Ascending colon cancer	IIIb	WallFlex™ Colonic Stent (22 × 120 mm)	None	3	4	0	2	4	3	Deceased
83	F	Pancreatic head cancer (portal vein invasion)	IV	WallFlex™ Duodenal Soft Stent (20 × 90 mm)	None	2	4	0	3	3	3	Deceased

This method effectively alleviated cancer-related obstructions. Adherence to appropriate indications and established guidelines was critical for ensuring the safe execution of the procedure.

## Conclusions

ESP provides an effective palliative option for cancer patients with intestinal obstruction when surgery is not feasible. This procedure allows the resumption of oral intake, alleviates symptoms of ileus, and leads to meaningful improvements in quality of life. The present cases demonstrate how ESP can support both clinical management and facilitate home discharge in advanced disease. Thus, ESP represents a practical and beneficial strategy for enhancing end-of-life quality in carefully selected patients.

## References

[1] Costa A, Yamashita ET, Takahashi W, Perosa M, Genzini T (2012). Malignant duodenal obstruction: palliative endoscopic treatment using self-expanding metal prosthesis [Article in Portuguese]. Rev Assoc Med Bras (1992).

[2] Vanella G, Coluccio C, Di Giulio E, Assisi D, Lapenta R (2019). Tertiary stent-in-stent for obstructing colorectal cancer: a case report and literature review. World J Gastrointest Endosc.

[3] Kaida T, Doi K, Yumoto S, Kinoshita S, Takeyama H, Ishiodori H, Baba H (2021). Cost-effectiveness of self-expandable metallic stents as bridge to surgery for obstructive colorectal cancer. Int J Clin Oncol.

[4] Nitta T, Fujii K, Hirata Y (2016). Reocclusion after self-expandable metallic stent placement for relieving malignant colorectal obstruction as a palliative treatment. Case Rep Gastroenterol.

[5] Ohta R, Sakon R, Goto M, Tachimori Y, Sekikawa K (2018). Self-expanding metal stent restenosis in obstructive colon diverticulitis mimicking colon cancer: a case report. Int J Surg Case Rep.

[6] Nitta T, Kataoka J, Ohta M (2017). Clinical outcomes of self-expandable metal stent (SEMS) placement as palliative treatment for malignant colorectal obstruction: a single-center study from Japan. Ann Med Surg (Lond).

[7] Inoue H, Arita T, Kuriu Y (2021). Emergency management of obstructive colorectal cancer - a retrospective study of efficacy and safety in self-expanding metallic stents and trans-anal tubes. In Vivo.

[8] Dohmoto M (1991). New method: endoscopic implantation of rectal stent in palliative treatment of malignant stenosis. Endosc Dig.

[9] van Hooft JE, Veld JV, Arnold D (2020). Self-expandable metal stents for obstructing colonic and extracolonic cancer: European Society of Gastrointestinal Endoscopy (ESGE) Guideline - Update 2020. Endoscopy.

[10] Ciambella CC, Beard RE, Miner TJ (2018). Current role of palliative interventions in advanced pancreatic cancer. World J Gastrointest Surg.

[11] Morris-Stiff G, Hassn A, Young WT (2008). Self-expanding metal stents for duodenal obstruction in advanced pancreatic adenocarcinoma. HPB (Oxford).

[12] Sakai A, Shiomi H, Okabe Y (2015). Effectiveness of endoscopic self-expandable metal stent placement for afferent loop obstruction caused by pancreatic cancer recurrence after pancreaticoduodenectomy. Clin J Gastroenterol.

[13] Due-Petersson R, Hansen LB (2021). Lumen-apposing metal stent for treatment of malignant biliary obstruction, placed through an uncovered duodenal self-expanding metal stent. BMJ Case Rep.

[14] Matsumoto K, Hayashi A, Yashima K (2014). Late complications of self-expandable metallic stent placement for malignant gastric outlet obstruction. Intern Med.

[15] Bulut E, Çiftçi T, Akhan O, Akıncı D (2017). Palliation of malignant gastroduodenal obstruction: fluoroscopic metallic stent placement with different approaches. Diagn Interv Radiol.

[16] Heo YC, Han DK, Kim MT (2021). Therapeutic effect of local photothermal heating of gold nanoparticle-coated self-expandable metallic stents for suppressing granulation tissue formation in the mouse colon. PLoS ONE.

[17] Matsuda A, Miyashita M, Matsumoto S (2019). Colonic stent-induced mechanical compression may suppress cancer cell proliferation in malignant large bowel obstruction. Surg Endosc.

[18] Delarue M, Montel F, Vignjevic D, Prost J, Joanny JF, Cappello G (2014). Compressive stress inhibits proliferation in tumor spheroids through a volume limitation. Biophys J.

[19] Saida Y (2016). Key points in the technique of colonic stent placement for malignant colorectal obstruction [Article in Japanese]. Gastroenterol Endosc.

[20] Sasaki T, Yoshida S, Isayama H, Koike K (2015). Metal stent treatment for malignant gastrointestinal obstruction - detention by type of stent [Article in Japanese]. Gastroenterol Endosc.

